# Risk-Adapted Management of Acute Pulmonary Embolism: Recent Evidence, New Guidelines

**DOI:** 10.5041/RMMJ.10174

**Published:** 2014-10-29

**Authors:** Anja Käberich, Simone Wärntges, Stavros Konstantinides

**Affiliations:** 1 Center for Thrombosis and Hemostasis (CTH), University Medical Center Mainz, Mainz, Germany; 2 Department of Cardiology, Democritus University of Thrace, Alexandroupolis, Greece

**Keywords:** Novel oral anticoagulants, pulmonary embolism, risk-adapted management, risk stratification, thrombolysis

## Abstract

Venous thromboembolism (VTE), the third most frequent acute cardiovascular syndrome, may cause life-threatening complications and imposes a substantial socio-economic burden. During the past years, several landmark trials paved the way towards novel strategies in acute and long-term management of patients with acute pulmonary embolism (PE). Risk stratification is increasingly recognized as a cornerstone for an adequate diagnostic and therapeutic management of the highly heterogeneous population of patients with acute PE. Recently published European Guidelines emphasize the importance of clinical prediction rules in combination with imaging procedures (assessment of right ventricular function) and laboratory biomarkers (indicative of myocardial stress or injury) for identification of normotensive PE patients at intermediate risk for an adverse short-term outcome. In this patient group, systemic full-dose thrombolysis was associated with a significantly increased risk of intracranial bleeding, a complication which discourages its clinical application unless hemodynamic decompensation occurs. A large-scale clinical trial program evaluating new oral anticoagulants in the initial and long-term treatment of venous thromboembolism showed at least comparable efficacy and presumably increased safety of these drugs compared to the current standard treatment. Research is continuing on catheter-directed, ultrasound-assisted, local, low-dose thrombolysis in the management of intermediate-risk PE.

## INTRODUCTION

Venous thromboembolism (VTE) is the third most frequent acute cardiovascular syndrome in industrialized countries, accounting for approximately 100 to 200 new cases per 100,000 population per year.^1^,^2^ As the incidence of VTE increases in an exponential manner with age, ongoing demographic changes will result in a growing number of patients suffering from the acute and long-term sequelae of VTE in the future.^3^ Approximately one-third of all patients with VTE present with acute pulmonary embolism (PE), with or without clinically evident deep vein thrombosis; acute PE accounts for the majority of VTE-associated hospitalizations and deaths.^2^ The broad spectrum of clinical presentations of PE ranges from clinically silent thromboembolic events to sudden death due to fulminant right ventricular failure. The non-specific signs and symptoms of acute PE frequently hamper diagnosis, resulting in an underestimation of the actual frequency of disease. This is supported by data derived from epidemiologic models suggesting that only 7% of patients dying early in the course of acute PE are diagnosed correctly during life.^2^ In unselected patients, case fatality rates in the acute phase range from 5% to 15%, and it has been calculated that as many as 370,000 deaths may be related to PE in Europe each year.^2^

This review elaborates on the risk-adapted diagnostic work-up and the acute-phase therapeutic management of patients with PE, highlighting recently published data and the revised guidelines and recommendations issued by the European Society of Cardiology (ESC) and endorsed by the European Respiratory Society (ERS). Particular focus is placed on the risk stratification of normotensive patients, the emerging role of new (non-vitamin K-dependent) oral anticoagulants for the treatment and secondary prophylaxis of acute PE, and the clinical benefits, risks, and indications of thrombolysis and other modes of reperfusion treatment.

## INITIAL RISK STRATIFICATION

Rational use of diagnostic procedures to confirm (or exclude) the presence of acute PE, and subsequent treatment decisions, should be based upon a reliable assessment of the patient’s risk of early mortality or other major cardiovascular complications. The presence and severity of right ventricular (RV) dysfunction is known to be a crucial determinant of outcome in the acute phase of PE.^4^,^5^ Functional impairment of the right ventricle is due to thromboembolic obstruction of the pulmonary arterial vasculature causing an acute increase of RV afterload which results in RV dilatation, increased wall tension, and RV ischemia, which in turn perpetuate hemodynamic worsening. Overall, less than 5% of patients with acute PE present with hemodynamic compromise (shock or persistent arterial hypertension) on admission due to clinically overt RV failure.^6^ This condition is associated with an estimated PE-related early mortality risk of at least 15%, a fact which mandates emergency advanced medical care.^7^ Thus, initial triage of patients with suspected acute PE should be based upon the assessment of the hemodynamic (clinical) stability allowing for a simplified classification into a high-risk or a non-high-risk group. This approach allows all subsequent diagnostic and therapeutic strategies to be adapted to the acuteness and severity of the clinical situation, maximizing efficiency of resource utilization and potentially saving lives.

## RISK-ADAPTED DIAGNOSTIC ALGORITHM

**Figure 1.**
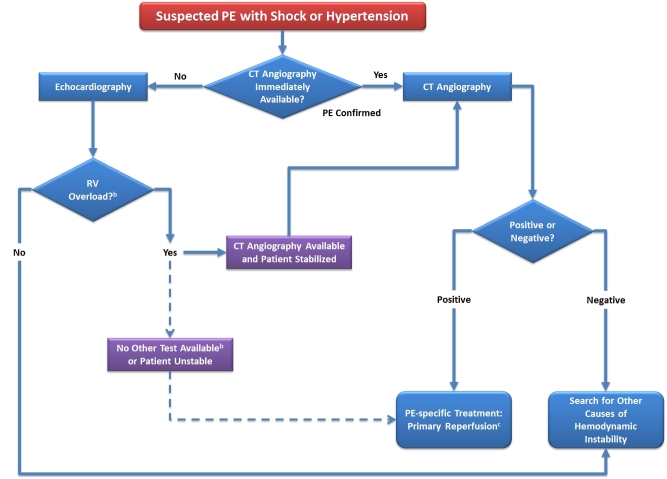
**Proposed Diagnostic Algorithm for Patients with Suspected High-Risk PE, i.e. Presenting with Shock or Hypotension.** ^a^ Includes the cases in which the patient’s condition is so critical that it only allows bedside diagnostic tests. ^b^ Apart from the diagnosis of RV dysfunction, bedside transthoracic echocardiography may, in some cases, directly confirm PE by visualizing mobile thrombi in the right heart chambers. ^c^ Thrombolysis; alternatively, surgical embolectomy or catheter-directed treatment. CT, computed tomographic (pulmonary angiography); PE, pulmonary embolism; RV, right ventricle.

**Figure 2.**
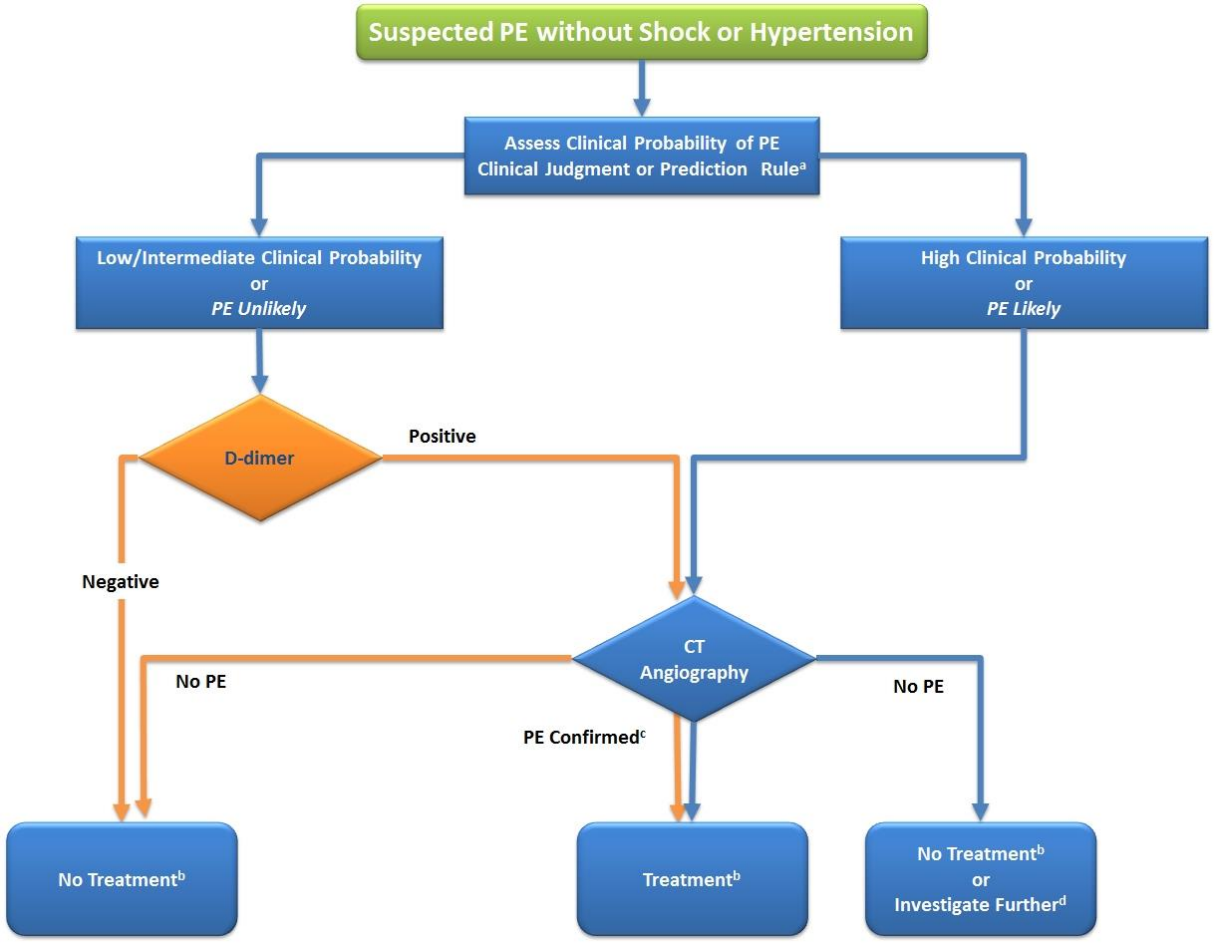
**Proposed Diagnostic Algorithm for Patients with Suspected High-Risk PE in the Absence of Shock or Hypotension.** ^a^ Two alternative classification schemes may be used for clinical probability assessment, i.e. a three-level scheme (clinical probability defined as low, intermediate, or high) or a two-level scheme (PE unlikely or PE likely). When using a moderately sensitive assay, D-dimer measurement should be restricted to patients with low clinical probability or a PE-unlikely classification, while highly sensitive assays may also be used in patients with intermediate clinical probability of PE due to a higher sensitivity and negative predictive value. ^b^ Treatment refers to anticoagulation treatment for PE. ^c^ CT angiogram is considered diagnostic of PE if it shows PE at the segmental or more proximal level. ^d^ In case of a negative CT angiogram in patients with high clinical probability, further investigation may be considered before withholding PE-specific treatment. CT, computed tomographic; PE, pulmonary embolism.

Based upon the initial stratification of patients into those with (suspected) high-risk PE either with or without shock or hypotension the ESC guidelines^8^ recommend two distinct algorithms (Figures 1 and 2, respectively) for diagnostic work-up. Clearly, however, diagnostic approaches may vary among hospitals depending on local expertise and the availability of individual imaging modalities.

### Suspected High-Risk PE with Shock or Hypotension (Recommended Algorithm Shown in Figure 1)

Suspected high-risk PE is an emergency situation. Multidetector computed tomographic pulmonary angiography (CTPA) is recommended, if immediately available, for confirmation of the diagnosis. Alternatively, transthoracic echocardiography performed as a bedside examination for detection of RV dysfunction indirectly confirms acute “massive” PE.^9^ Apart from verifying RV dysfunction (indicated by RV dilatation, paradoxical septal movement, abnormal motion of the RV free wall (McConnell sign), disturbed RV ejection pattern, triscuspid valve regurgitation, and dilatation and missing inspiratory collapse of the inferior vena cava) and pulmonary hypertension (increased tricuspid regurgitant jet velocity and/or pulmonary arterial dilatation),^8^ transthoracic echocardiography may indicate the presence of mobile thrombi in the right-sided heart cavities.^10^–^12^ In unstable patients, signs of RV dysfunction on echocardiography are sufficient for prompt initiation of reperfusion therapy (e.g. systemic thrombolysis) without the necessity of further testing. Moreover, echocardiography may help to detect or exclude alternative causes of shock such as aortic dissection, pericardial tamponade, or severe left ventricular failure. In patients primarily admitted to the catheterization laboratory to diagnose or exclude an acute coronary syndrome, pulmonary angiography can be considered as an alternative diagnostic approach.^8^

### Suspected High-Risk PE Without Shock or Hypotension (Recommended Algorithm Shown in Figure 2)

Pulmonary embolism may escape prompt diagnosis in hemodynamically stable patients, since clinical signs and symptoms such as dyspnea, chest pain, (pre-) syncope, or hemoptysis are frequently absent or, if present, non-specific.^13^–^17^ In this group of “stable” patients, diagnostic certainty is the physician’s first priority in order to prevent VTE recurrence but also to avoid unnecessary long-term anticoagulation which may, by itself, cause potentially life-threatening complications. The diagnostic strategy should begin with assessment of the clinical probability of PE using either validated explicit clinical prediction rules or implicit clinical judgment. In the past years, simplified versions of the revised Geneva prediction rule^18^ and the Wells score,^19^ both assessing the pre-test probability of acute PE, were developed and externally validated.^20^,^21^ Based on either the original or the simplified versions of these prediction rules,^20^,^22^–^33^ D-dimer testing is indicated as a second diagnostic step before performing an imaging test in patients with low or intermediate clinical probability of PE (or PE-unlikely using a dichotomized score), whereas in cases with high clinical probability (or PE-likely), it is recommended to proceed to imaging procedures without laboratory testing.^8^

Plasma concentrations of D-dimers are elevated in acute thrombus formation due to simultaneous stimulation of the fibrinolytic cascade and formation of fibrin cleavage products. As the sensitivity and negative predictive value of ELISA-based D-dimer assays are high,^34^,^35^ PE can be safely ruled out in patients with low or intermediate clinical probability of the disease and a negative D-dimer test. These patients can be left untreated (i.e. without anticoagulation), as proven in outcome studies and a meta-analysis which indicated a 3-month thromboembolic risk below 1%.^36^–^41^ Notably, in hospitalized patients, D-dimer measurement is of limited use. On the other hand, it is well known that the specificity of D-dimer testing (∼30%^42^) is low and thus by no means confirms the disease but only indicates that further testing for PE is necessary. Several comorbidities or concomitant conditions, such as active malignancy,^43^,^44^ hospitalization due to other causes,^20^,^45^ postoperative state,^46^ pregnancy,^47^,^48^ or “simply” advanced age,^42^ can induce non-specific increases in D-dimer plasma levels. As recently shown, age-adjusted D-dimer cut-off levels (age × 10 µg/L for patients above 50 years) rather than the established fixed cut-off of 500 µg/L may help increase the specificity of D-dimer measurements;^49^ in a multicenter management study, the proportion of patients in whom acute PE could be excluded without further testing increased to approximately 30% without elevating the numbers of false-negative findings.^49^

In all patients with a high clinical probability for acute PE, and in those with a positive D-dimer test, CTPA visualizing at least one clot at the segmental or more proximal level of the pulmonary arteries confirms PE with high sensitivity (83%) and specificity (96%);^50^ alternatively, CTPA may help establish an alternative diagnosis. Uncertainty persists with regard to the clinical significance of isolated subsegmental pulmonary emboli which were confirmed in 4.7% of patients imaged by single-detector CT and in 9.4% assessed with multi-detector CTPA.^51^ Poor interobserver agreement and the low positive predictive value of such findings justify further testing (e.g. with compression ultrasound) to confirm PE in this specific setting.^8^

Although CTPA has largely replaced other imaging modalities in the diagnosis of acute PE, a ventilation/perfusion lung scan remains a valuable, radiation- and contrast medium-sparing diagnostic option, especially for patients with contraindications to CT imaging (including those with severe renal insufficiency, hyperthyroidism, or contrast medium allergy) or in order to avoid unnecessary radiation in younger female patients as well as in pregnant or breast-feeding women. Compression ultrasound sonography visualizing proximal deep vein thrombosis also confirms PE without the need for further imaging tests.

## FURTHER RISK STRATIFICATION OF NORMOTENSIVE PATIENTS WITH PE

Prognostic assessment of confirmed acute PE is based upon the patient’s individual risk of early mortality, taking into consideration the clinical severity of PE as well as the patient’s cardiopulmonary reserves and concomitant co-morbidities. As already mentioned, high-risk PE is characterized by overt hemodynamic compromise (cardiogenic shock or persistent arterial hypotension); this emergency situation demands immediate confirmation of the diagnosis and treatment to save the patient’s life. In hemodynamically stable (non-high-risk) patients with confirmed PE, advanced risk stratification intends to identify either patients at low risk for early (usually 30-day) mortality, who may be suitable for early discharge and home treatment, or patients at an intermediate risk who may benefit from advanced medical care, monitoring, and possibly early reperfusion therapy. Prognostic assessment should use a validated clinical prognostic score such as the Pulmonary Embolism Severity Index (PESI)^52^ or its simplified version (sPESI)^53^ (Table 1). Patients with a sPESI score of ≥1 point(s) or a PESI class of III–IV represent approximately two-thirds of unselected PE patients and are characterized by a 30-day mortality rate of 11%–25%.^52^,^53^ These patients are considered to have *intermediate-risk* PE.^8^ In this subgroup, further risk assessment consisting of cardiac biomarker levels (such as, for myocardial injury, cardiac troponins I or T; or, for cardiac failure, natriuretic peptides), and the presence of RV dysfunction on CT or echocardiography should be considered. This enables patient classification into either *intermediate–low* (RV dysfunction present *or* cardiac biomarker levels elevated *or* none of the two present) or *intermediate–high* risk (presence of RV dysfunction *plus* elevated cardiac biomarker levels), which will guide further treatment decisions.

**Table 1.** Original and Simplified Pulmonary Embolism Severity Index.

Parameter	Original Version^52^	Simplified Version^53^
Age	Age in years	1 point (if age >80 years)
Male sex	+10 points	–
Cancer	+30 points	1 point
Chronic heart failure	+10 points	1 point
Chronic pulmonary disease	+10 points	
Pulse rate ≥110 bpm	+20 points	1 point
Systolic BP <100 mmHg	+30 points	1 point
Respiratory rate >30 breaths per minute	+20 points	–
Temperature <36°C	+20 points	–
Altered mental status	+60 points	–
Arterial oxyhemoglobin saturation <90%	+20 points	1 point
Risk strata^a^		
	**Class I:** ≤65 points—very low 30-day mortality risk (0% to 1.6%)	0 points—30-day mortality risk 1.0% (95% CI 0.0%–2.1%)
	**Class II:** 66–85 points—low mortality risk (1.7%–3.5%)	
	_____________	
	**Class III:** 86–105 points—moderate mortality risk (3.2%–7.1%)	
	**Class IV:** 106–125 points—high mortality risk (4.0%–11.4%)	≥1 point(s)—30-day mortality risk 10.9% (95% CI 8.5%–13.2%)
	**Class V:** >125 points—very high mortality risk (10.0%–24.5%)	

a Based on the sum of points.

bpm, beats per minute; PESI, pulmonary embolism severity index.

In patients having a low risk of 30-day mortality according to the sPESI or the PESI, additional prognostic assessment using laboratory marker tests of or the evaluation of RV function by imaging modalities is not deemed necessary. However, if one or both of these tests have already been performed on admission, before (s)PESI calculation, and yielded abnormal findings (a sequence of events which is quite likely in clinical routine), then patients should probably also be classified into the *intermediate–low-risk* category and treated as explained below.

## ACUTE-PHASE MANAGEMENT OF THE PULMONARY EMBOLISM

### Anticoagulation

Anticoagulation prevents both early death and recurrent symptomatic or fatal VTE. The standard duration of anticoagulation should cover at least 3 months. Within this period, acute-phase treatment consists of parenteral anticoagulation (unfractionated heparin, low-molecular-weight heparin (LMWH), or fondaparinux) administration over the first 5–10 days. Parenteral heparin administration should overlap with the initiation of a vitamin K antagonist (VKA), or it can be followed by one of the new oral anticoagulants dabigatran or edoxaban (see below). If rivaroxaban or apixaban is given instead (see below for studies), oral treatment with one of these agents should be started directly or after a 1–2-day administration of unfractionated heparin, LMWH, or fondaparinux. In this latter case, acute-phase treatment consists of an increased dose over the first 3 weeks (for rivaroxaban), or over the first 7 days (for apixaban).

The non-vitamin K-dependent new oral anticoagulants, i.e. the direct thrombin inhibitor dabigatran and the direct factor Xa inhibitors rivaroxaban, apixaban, and edoxaban, have been tested in large phase 3 clinical trials. In RE-COVER I and II, dabigatran was compared with warfarin for the treatment of VTE. The primary outcome was the 6-month incidence of recurrent symptomatic or fatal VTE. In the pooled analysis of the results of the “twin” studies RECOVER I and II, including a total of 5,109 patients,^54^ dabigatran was non-inferior to warfarin with regard to the primary efficacy endpoint (observed incidence 2.4% versus 2.2%; HR 1.09, 95% CI 0.76–1.57). Major bleeding appeared to occur with lower frequency in the dabigatran group, both during the period starting at first intake of study drug (which included the initial warfarin loading together with heparin treatment in the control arm as opposed to heparin alone until the switch to the oral anticoagulant in the dabigatran arm; HR 0.73 for dabigatran, 95% CI 0.48–1.11) and during the double-dummy phase (comparing monotherapy of dabigatran versus warfarin; HR 0.60, 95% CI 0.36–0.99).

In the EINSTEIN-DVT^55^ and EINSTEIN-PE^56^ trials, single oral drug treatment with the direct factor Xa inhibitor rivaroxaban was tested in patients with VTE using a randomized, open-label, non-inferiority design. In the pooled analysis of the results of both studies, including a total of 8,282 patients,^57^ rivaroxaban was non-inferior to standard therapy for the primary efficacy outcome (observed incidence 2.1% versus 2.3%; HR 0.89, 95% CI 0.66–1.19). Major bleeding occurred with lower frequency in the rivaroxaban group (HR 0.54, 95% CI 0.37–0.79).

The Apixaban for the Initial Management of Pulmonary Embolism and Deep-Vein Thrombosis as First-line Therapy (AMPLIFY) study compared single oral drug treatment with apixaban with standard therapy in 5,395 patients with acute VTE.^58^ Apixaban was non-inferior to conventional treatment for the primary efficacy, and major bleeding occurred less frequently under apixaban compared with standard therapy. A significant difference in favor of apixaban was also observed for the composite outcome of major or clinically relevant non-major bleeding (observed incidence 4.3% versus 9.7%; RR 0.44, 95% CI 0.36–0.55).

Finally, the Hokusai-VTE trial compared edoxaban with conventional therapy in 8,240 patients with VTE who had initially received heparin for at least 5 days.^59^ Patients received edoxaban at a dose of 60 mg once daily (reduced to 30 mg once daily in the case of creatinine clearance of 30–50 mL/min or a body weight <60 kg), or warfarin. In contrast to the fixed anticoagulation period(s) followed in previous trials, the study drug was administered for 3–12 months based on the investigators’ judgment; all patients were followed for 12 months. Edoxaban was non-inferior to warfarin with respect to the primary efficacy outcome of recurrent symptomatic VTE. Major bleeding or clinically relevant non-major bleeding was less frequently observed in the edoxaban group (HR 0.81, 95% CI 0.71–0.94).

Taken together, the results of the trials using new oral anticoagulants in the treatment of VTE indicate that these agents are at least as effective and probably safer (in terms of major bleeding) than the standard heparin/VKA regimen. Experience with the handling of these drugs in different clinical scenarios, and with the management of their bleeding complications, continues to accumulate, and useful practical recommendations have recently been published by the European Heart Rhythm Association.^60^ Currently, rivaroxaban, dabigatran, and apixaban are approved for treatment of VTE in Europe.

### Thrombolytic, Interventional, or Surgical Treatment

In unstable patients with high-risk PE, large-scale epidemiological data support the notion that inhospital mortality can be lowered by thrombolytic treatment.^6^ Therefore, thrombolysis is recommended as first-line therapy in this patient group. Surgical and interventional treatments represent alternative options, particularly if the bleeding risk under thrombolysis is considered to be high and provided that the necessary infrastructure, equipment, and expertise are available on site.

In non-high-risk PE, the clinical benefits of thrombolysis have remained controversial for many years.^61^ Recently, a large multicenter, randomized trial compared, in a double-blind manner, thrombolysis with tenecteplase plus heparin versus placebo plus heparin in 1,006 patients with intermediate-risk PE.^62^ Patients had RV dysfunction confirmed by echocardiography or CT angiography, and myocardial injury confirmed by a positive troponin I or T test. The primary efficacy outcome, a composite of all-cause death or hemodynamic decompensation/collapse within 7 days of randomization, was significantly reduced with tenecteplase (2.6% versus 5.6% in the placebo group; OR 0.44, 95% CI 0.23–0.88). The clinical benefit was driven mainly by a significant reduction in the rate of hemodynamic collapse (1.6% versus 5.0%, *P* = 0.002), while all-cause mortality was low, both in the tenecteplase and in the placebo group (1.2% versus 1.8%; *P* = 0.43).^62^ On the other hand, the safety data were not favorable for thrombolysis, as the trial demonstrated a 2% risk of hemorrhagic stroke after thrombolytic treatment with tenecteplase; major non-intracranial bleeding events were also increased in the tenecteplase compared with the placebo group (6.3% versus 1.5%; *P*<0.001).^62^ These results indicate that routine primary thrombolysis is not recommended for normotensive patients with acute PE, unless they show clinical signs of hemodynamic decompensation.

An alternative to systemic full-dose thrombolysis may consist of local, catheter-delivered, ultrasound-assisted thrombolysis using small doses of a thrombolytic agent, provided of course that the necessary infrastructure, equipment, and expertise are all available on site. In a phase 2 clinical trial, 59 patients with acute main- or lower-lobe PE and echocardiographic right-to-left ventricular dimension ratio ≥1.0 were randomized to receive unfractionated heparin and an ultrasound-assisted thrombolytic regimen of 10–20 mg recombinant tissue plasminogen activator (rtPA) plus unfractionated heparin over 15 hours as opposed to unfractionated heparin alone. Reduced-dose local thrombolysis significantly reduced, compared to heparin alone, the subannular right-to-left ventricular dimension ratio from baseline to 24 hours without an increase in bleeding complications.^63^ The efficacy and safety of local, “pharmacomechanical” thrombolysis is also supported by the results of a recently presented prospective, single-arm multicenter trial which enrolled 150 patients with submassive or massive PE (Clinicaltrials.gov identifier: NCT01513759).

## CONCLUSIONS

**Figure 3.**
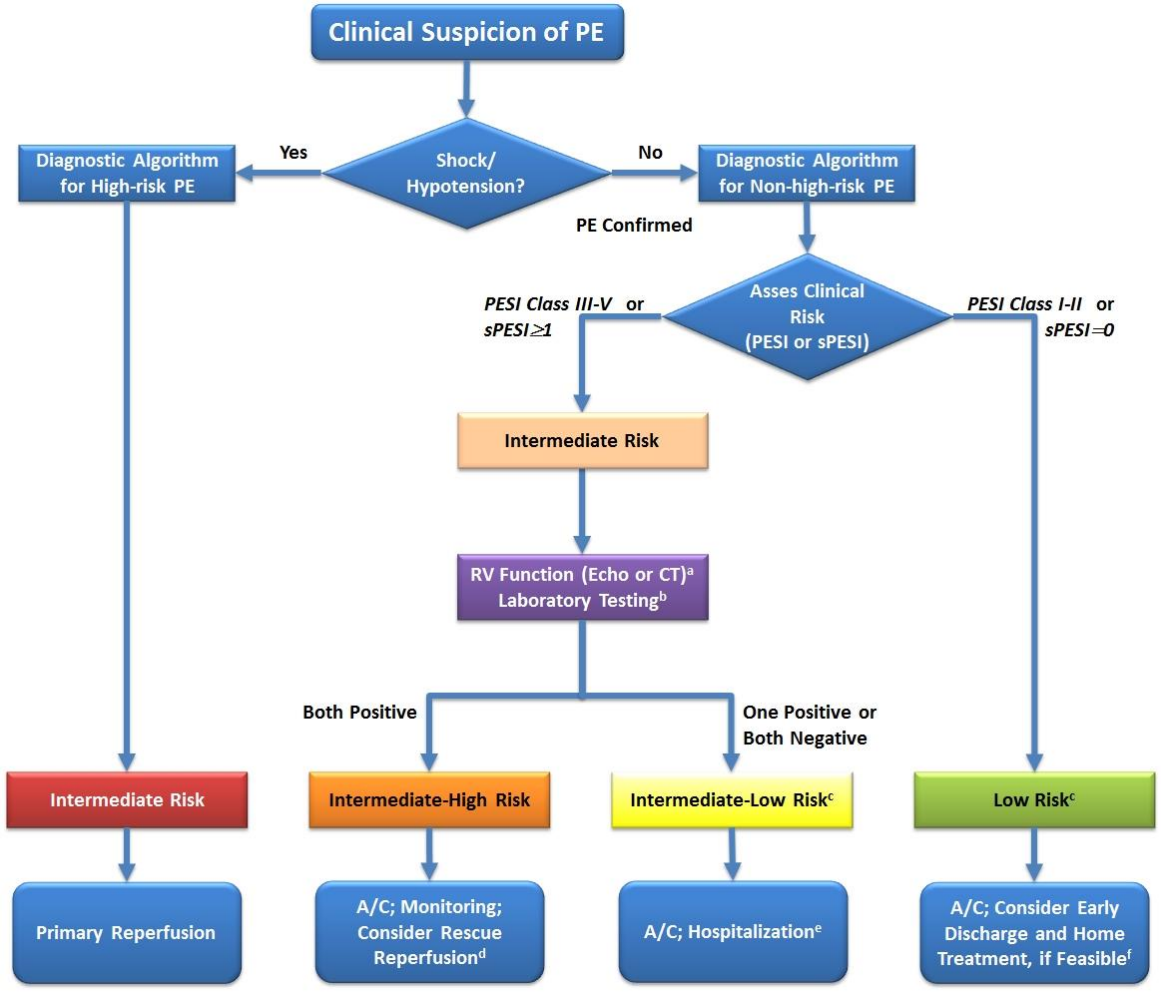
**Risk-Adjusted Management Strategies in Acute PE. Based on Konstantinides et al.^8^** ^a^ If echocardiography has already been performed during diagnostic work-up for PE and detected RV dysfunction, or if the CT already performed for diagnostic work-up has shown RV enlargement (right/left ventricular ratio ≥0.9), a cardiac troponin test should be performed except for cases in which primary reperfusion is not a therapeutic option (e.g. due to severe co-morbidity or limited life expectancy of the patient). ^b^ Markers of myocardial injury (e.g. elevated cardiac troponin I or -T concentrations in plasma) or of heart failure as a result of (right) ventricular dysfunction (elevated natriuretic peptide concentrations in plasma). If a laboratory test for a cardiac biomarker has already been performed during initial diagnostic work-up (e.g. in the chest pain unit) and was positive, then an echocardiogram should be considered to assess RV function, or RV size should be (re)assessed on CT. ^c^ Patients in the PESI Class I–II, or with sPESI of 0, and elevated cardiac biomarkers or signs of RV dysfunction on imaging tests are also to be classified into the intermediate-to-low-risk category. This might apply to situations in which imaging or biomarker results become available before calculation of the clinical severity index. These patients are probably not candidates for home treatment. ^d^ Thrombolysis, if (and as soon as) clinical signs of hemodynamic decompensation appear; surgical pulmonary embolectomy or percutaneous catheter-directed treatment may be considered as alternative options to systemic thrombolysis, particularly if the bleeding risk is high. ^e^ Monitoring should be considered for patients with confirmed PE and a positive troponin test, even if there is no evidence of RV dysfunction on echocardiography or CT. ^f^ The simplified version of the PESI has not been validated in prospective home treatment trials; inclusion criteria other than the PESI were used in two single-armed (non-randomized) management studies. A/C, anticoagulation; CT, computed tomographic pulmonary angiography; PE, pulmonary embolism; PESI, pulmonary embolism severity index; RV, right ventricular; sPESI, simplified pulmonary embolism severity index.

Venous thromboembolism has received relatively little attention from the scientific and medical community for decades. Recently, however, advances in diagnostic imaging, along with the development of new antithrombotic agents and strategies, increased awareness of the importance of VTE and began to improve patient outcomes in the acute phase and over the long term. The new evidence that accumulated in all these areas has led to clear-cut, clinical practice-relevant recommendations which are included in the recently updated ESC Guidelines on the management of acute pulmonary embolism (Figure 3).^8^

## Abbreviations:

CTPA: computed tomographic pulmonary angiography;
DVT: deep vein thrombosis;
INR: international normalized ratio;
LMWH: low-molecular-weight heparin;
NOAC: new (non-vitamin K-dependent) oral anticoagulant(s);
NT-proBNP: N-terminal propeptide of brain natriuretic peptide;
PE: pulmonary embolism;
(s)PESI: (simplified) pulmonary embolism severity index;
PPV: positive predictive value;
rtPA: recombinant tissue plasminogen activator;
RV: right ventricle/right ventricular;
VKA: vitamin K antagonists;
VTE: venous thromboembolism.

## References

[1] Heit JA (2008). The epidemiology of venous thromboembolism in the community. Arterioscler Thromb Vasc Biol.

[2] Cohen AT, Agnelli G, Anderson FA (2007). Venous thromboembolism (VTE) in Europe. The number of VTE events and associated morbidity and mortality. Thromb Haemost.

[3] Anderson FA, Spencer FA (2003). Risk factors for venous thromboembolism. Circulation.

[4] Lualdi JC, Goldhaber SZ (1995). Right ventricular dysfunction after acute pulmonary embolism: pathophysiologic factors, detection, and therapeutic implications. Am Heart J.

[5] Konstantinides S (2005). Pulmonary embolism: impact of right ventricular dysfunction. Curr Opin Cardiol.

[6] Stein PD, Matta F (2012). Thrombolytic therapy in unstable patients with acute pulmonary embolism: saves lives but underused. Am J Med.

[7] Laporte S, Mismetti P, Decousus H (2008). Clinical predictors for fatal pulmonary embolism in 15,520 patients with venous thromboembolism: findings from the Registro Informatizado de la Enfermedad TromboEmbolica venosa (RIETE) Registry. Circulation.

[8] Konstantinides S, Torbicki A, Agnelli G (2014). ESC Guidelines on the diagnosis and management of acute pulmonary embolism: The Task Force for the Diagnosis and Management of Acute Pulmonary Embolism of the European Society of Cardiology (ESC) Endorsed by the European Respiratory Society (ERS). Eur Heart J.

[9] Kucher N, Luder CM, Dornhofer T, Windecker S, Meier B, Hess OM (2003). Novel management strategy for patients with suspected pulmonary embolism. Eur Heart J.

[10] Mansencal N, Attias D, Caille V (2011). Computed tomography for the detection of free-floating thrombi in the right heart in acute pulmonary embolism. Eur Radiol.

[11] Torbicki A, Galie N, Covezzoli A, Rossi E, De Rosa M, Goldhaber SZ (2003). Right heart thrombi in pulmonary embolism: results from the International Cooperative Pulmonary Embolism Registry. J Am Coll Cardiol.

[12] Casazza F, Bongarzoni A, Centonze F, Morpurgo M (1997). Prevalence and prognostic significance of right-sided cardiac mobile thrombi in acute massive pulmonary embolism. Am J Cardiol.

[13] Miniati M, Prediletto R, Formichi B (1999). Accuracy of clinical assessment in the diagnosis of pulmonary embolism. Am J Respir Crit Care Med.

[14] Pollack CV, Schreiber D, Goldhaber SZ (2011). Clinical characteristics, management, and outcomes of patients diagnosed with acute pulmonary embolism in the emergency department: initial report of EMPEROR (Multicenter Emergency Medicine Pulmonary Embolism in the Real World Registry). J Am Coll Cardiol.

[15] Wells PS, Ginsberg JS, Anderson DR (1998). Use of a clinical model for safe management of patients with suspected pulmonary embolism. Ann Intern Med.

[16] Thames MD, Alpert JS, Dalen JE (1977). Syncope in patients with pulmonary embolism. JAMA.

[17] Stein PD, Henry JW (1997). Clinical characteristics of patients with acute pulmonary embolism stratified according to their presenting syndromes. Chest.

[18] Klok FA, Mos IC, Nijkeuter M (2008). Simplification of the revised Geneva score for assessing clinical probability of pulmonary embolism. Arch Intern Med.

[19] Gibson NS, Sohne M, Kruip MJ (2008). Further validation and simplification of the Wells clinical decision rule in pulmonary embolism. Thromb Haemost.

[20] Douma RA, Mos IC, Erkens PM (2011). Performance of 4 clinical decision rules in the diagnostic management of acute pulmonary embolism: a prospective cohort study. Ann Intern Med.

[21] Douma RA, Gibson NS, Gerdes VE (2009). Validity and clinical utility of the simplified Wells rule for assessing clinical probability for the exclusion of pulmonary embolism. Thromb Haemost.

[22] Le Gal G, Righini M, Roy PM (2006). Prediction of pulmonary embolism in the emergency department: the revised Geneva score. Ann Intern Med.

[23] Ceriani E, Combescure C, Le Gal G (2010). Clinical prediction rules for pulmonary embolism: a systematic review and meta-analysis. J Thromb Haemost.

[24] Lucassen W, Geersing GJ, Erkens PM (2011). Clinical decision rules for excluding pulmonary embolism: a meta-analysis. Ann Intern Med.

[25] Wells PS, Anderson DR, Rodger M (2000). Derivation of a simple clinical model to categorize patients probability of pulmonary embolism: increasing the models utility with the SimpliRED D-dimer. Thromb Haemost.

[26] Anderson DR, Kovacs MJ, Dennie C (2005). Use of spiral computed tomography contrast angiography and ultrasonography to exclude the diagnosis of pulmonary embolism in the emergency department. J Emerg Med.

[27] Kearon C, Ginsberg JS, Douketis J (2006). An evaluation of D-dimer in the diagnosis of pulmonary embolism: a randomized trial. Ann Intern Med.

[28] Sohne M, Kamphuisen PW, van Mierlo PJ, Buller HR (2005). Diagnostic strategy using a modified clinical decision rule and D-dimer test to rule out pulmonary embolism in elderly in- and outpatients. Thromb Haemost.

[29] van Belle A, Buller HR, Huisman MV (2006). Effectiveness of managing suspected pulmonary embolism using an algorithm combining clinical probability, D-dimer testing, and computed tomography. JAMA.

[30] Wells PS, Anderson DR, Rodger M (2001). Excluding pulmonary embolism at the bedside without diagnostic imaging: management of patients with suspected pulmonary embolism presenting to the emergency department by using a simple clinical model and d-dimer. Ann Intern Med.

[31] Rodger MA, Maser E, Stiell I, Howley HE, Wells PS (2005). The interobserver reliability of pretest probability assessment in patients with suspected pulmonary embolism. Thromb Res.

[32] Runyon MS, Webb WB, Jones AE, Kline JA (2005). Comparison of the unstructured clinician estimate of pretest probability for pulmonary embolism to the Canadian score and the Charlotte rule: a prospective observational study. Acad Emerg Med.

[33] Wolf SJ, McCubbin TR, Feldhaus KM, Faragher JP, Adcock DM (2004). Prospective validation of Wells Criteria in the evaluation of patients with suspected pulmonary embolism. Ann Emerg Med.

[34] Di Nisio M, Squizzato A, Rutjes AW, Buller HR, Zwinderman AH, Bossuyt PM (2007). Diagnostic accuracy of D-dimer test for exclusion of venous thromboembolism: a systematic review. J Thromb Haemost.

[35] Stein PD, Hull RD, Patel KC (2004). D-dimer for the exclusion of acute venous thrombosis and pulmonary embolism: a systematic review. Ann Intern Med.

[36] Perrier A, Roy PM, Aujesky D (2004). Diagnosing pulmonary embolism in outpatients with clinical assessment, D-dimer measurement, venous ultra-sound, and helical computed tomography: a multi-center management study. Am J Med.

[37] Perrier A, Roy PM, Sanchez O (2005). Multidetectorrow computed tomography in suspected pulmonary embolism. N Engl J Med.

[38] Perrier A, Desmarais S, Miron MJ (1999). Non-invasive diagnosis of venous thromboembolism in outpatients. Lancet.

[39] Kruip MJ, Slob MJ, Schijen JH, van der Heul C, Buller HR (2002). Use of a clinical decision rule in combination with D-dimer concentration in diagnostic work-up of patients with suspected pulmonary embolism: a prospective management study. Arch Intern Med.

[40] Righini M, Le Gal G, Aujesky D (2008). Diagnosis of pulmonary embolism by multidetector CT alone or combined with venous ultrasonography of the leg: a randomised non-inferiority trial. Lancet.

[41] Carrier M, Righini M, Djurabi RK (2009). VIDAS D-dimer in combination with clinical pre-test probability to rule out pulmonary embolism. A systematic review of management outcome studies. Thromb Haemost.

[42] Righini M, Goehring C, Bounameaux H, Perrier A (2000). Effects of age on the performance of common diagnostic tests for pulmonary embolism. Am J Med.

[43] Di Nisio M, Sohne M, Kamphuisen PW, Buller HR (2005). DDimer test in cancer patients with suspected acute pulmonary embolism. J Thromb Haemost.

[44] Righini M, Le Gal G, De Lucia S (2006). Clinical usefulness of D-dimer testing in cancer patients with suspected pulmonary embolism. Thromb Haemost.

[45] Miron MJ, Perrier A, Bounameaux H (1999). Contribution of noninvasive evaluation to the diagnosis of pulmonary embolism in hospitalized patients. Eur Respir J.

[46] Dindo D, Breitenstein S, Hahnloser D (2009). Kinetics of D-dimer after general surgery. Blood Coagul Fibrinolysis.

[47] Chabloz P, Reber G, Boehlen F, Hohlfeld P, de Moerloose P (2001). TAFI antigen and D-dimer levels during normal pregnancy and at delivery. Br J Haematol.

[48] Francalanci I, Comeglio P, Liotta AA (1995). D-dimer concentrations during normal pregnancy, as measured by ELISA. Thromb Res.

[49] Righini M, Van Es J, den Exter PL (2014). Age-adjusted D-dimer cutoff levels to rule out pulmonary embolism: the ADJUST-PE study. JAMA.

[50] Stein PD, Fowler SE, Goodman LR (2006). Multi-detector computed tomography for acute pulmonary embolism. N Engl J Med.

[51] Carrier M, Righini M, Wells PS (2010). Subsegmental pulmonary embolism diagnosed by computed tomography: incidence and clinical implications. A systematic review and meta-analysis of the management outcome studies. J Thromb Haemost.

[52] Aujesky D, Obrosky DS, Stone RA (2005). Derivation and validation of a prognostic model for pulmonary embolism. Am J Respir Crit Care Med.

[53] Jimenez D, Aujesky D, Moores L (2010). Simplification of the pulmonary embolism severity index for prognostication in patients with acute symptomatic pulmonary embolism. Arch Intern Med.

[54] Schulman S, Kakkar AK, Goldhaber SZ (2014). Treatment of acute venous thromboembolism with dabigatran or warfarin and pooled analysis. Circulation.

[55] Bauersachs R, Berkowitz SD, Brenner B (2010). Oral rivaroxaban for symptomatic venous thromboembolism. N Engl J Med.

[56] Buller HR, Prins MH, Lensin AW (2012). Oral rivaroxaban for the treatment of symptomatic pulmonary embolism. N Engl J Med.

[57] Prins MH, Lensing AW, Bauersachs R (2013). Oral rivaroxaban versus standard therapy for the treatment of symptomatic venous thromboembolism: a pooled analysis of the EINSTEIN-DVT and PE randomized studies. Thromb J.

[58] Agnelli G, Buller HR, Cohen A (2013). Oral apixaban for the treatment of acute venous thromboembolism. N Engl J Med.

[59] Buller HR, Decousus H, Grosso MA (2013). Edoxaban versus warfarin for the treatment of symptomatic venous thromboembolism. N Engl J Med.

[60] Heidbuchel H, Verhamme P, Alings M (2013). EHRA practical guide on the use of new oral anticoagulants in patients with non-valvular atrial fibrillation: executive summary. Eur Heart J.

[61] Konstantinides S, Geibel A, Heusel G, Heinrich F, Kasper W (2002). Heparin plus alteplase compared with heparin alone in patients with submassive pulmonary embolism. N Engl J Med.

[62] Meyer G, Vicaut E, Danays T (2014). Fibrinolysis for patients with intermediate-risk pulmonary embolism. N Engl J Med.

[63] Kucher N, Boekstegers P, Muller OJ (2014). Randomized, controlled trial of ultrasound-assisted catheter-directed thrombolysis for acute intermediate-risk pulmonary embolism. Circulation.

